# Inflammation of the Iris

**Published:** 1910-10-08

**Authors:** A. Maitland Ramsay

**Affiliations:** Ophthalmic Surgeon, Glasgow Royal Infirmary, and Lecturer on Eye Diseases, Queen Margaret College, University of Glasgow.


### Hospital Clinics.

### INFLAMMATION OF THE IRIS.

By A. MAITLAND RAMSAY, M.D., F.R.F.P.S.G., Ophthalmic Surgeon, Glasgow Royal Infirmary, and Lecturer on Eye Diseases, Queen Margaret College, University of Glasgow.

(A Clinical Lecture delivered in the Glasgow Ophthalmic Institution on June 16, 1910.)

Ladies and Gentlemen,—The four patients here, whose cases I am now to discuss, were admitted to my wards during the past week. They are all suffering from inflammation of the iris, and I wish to utilise them this afternoon for the purpose of emphasising the fundamental truth that in every idiopathic inflammation there is a predisposing as well as an exciting cause. The predisposing cause may, in some cases, be readily discoverable, and give the inflammation a character so distinctive as to be recognised at the bedside without difficulty; but it is, in other cases, quite unknown to the patient, and far from apparent on the surface. It must then be sought for with tact, caution, care, and perseverance, for on its proper recognition depends the success or the failure of treatment. It is true that in every case of iritis the indications for the local treatment are similar—to dilate the pupil and to relieve pain. If, however, recovery is to be satisfactory and relapse prevented, the constitutional state underlying the ocular inflammation must also be remedied. Every one of these four patients will say that the inflammation of the eye is the result of exposure to cold, but in each of them the natural history of the disease has been considerably modified by the condition of the general health. In all the cases atropine is the beginning and the end of local treatment, and the action of the mydriatic must be aided by every means at one's disposal for the relief of pain, but cure, in the proper sense of the word, is not possible unless equally energetic measures be taken against the diathesis or the infection which has been the predisposing cause.

### A Case of Catarrhal Conjunctivitis.

The first patient is a woman of thirty-six years of age, who was admitted to the Ophthalmic Institution on June 10, 1910. She says that about a year before that time she suffered from her first attack of conjunctivitis. She seems not to have made a proper recovery, for every now and then during the intervening twelve months she has suffered from red and watery eyes, and the eyelids have been glued together in the morning after sleep. She says that as a rule she enjoys good health, but that three years ago she had an attack of "inflammation of the kidneys," which, however, as far as can be made out, appears to have been nothing worse than renal colic. For at least three months before admission she had been much troubled by severe head-pain (mostly frontal and temporal), and she was conscious that she was much more nervous and excitable than she had formerly been. The present attack of inflammation of the eyes began about a week before she came to the Ophthalmic Institution, and was, from the onset, characterised by severe pain both in eyes and in head, the suffering being more acute on the right side than on the left. When she was admitted, not only was there pain, but the eyes watered freely and were very intolerant of light. There was injection of both palpebral and ocular conjunctiva, and the edges of the lids as well as both outer and inner canthi were smeared with catarrhal discharge, which on bacteriological examination was found to contain the Morax-Axenfeld diplobacillus. More careful examination revealed that in the right eye a deep pericorneal injection complicated the superficial conjunctival vascularity, and that the right iris was markedly discoloured when compared with the left. The right pupil was contracted, and dilated irregularly after the instillation of atropine; and in this eye the vision was misty, though the acuity was not much below normal. The refraction of the eyes is hypermetropic and astigmatic.

Here then we have to deal with a case of catarrhal conjunctivitis, which is complicated by inflammation of the iris of the right side. The danger is that the iritis might have been overlooked, and the case treated by a sulphate of zinc lotion, which is, as you know, a specific against the Morax-Axenfeld diplobacillus. The consequence of such an error would have been that the treatment, so useful for the cure of the conjunctivitis, would have aggravated the inflammation of the deep structures. When, therefore, one is dealing with conjunctivitis in an adult, the colour of the iris and the mobility of the pupil should be carefully examined before an irritant or astringent lotion is prescribed. That is a clinical rule which must never be forgotten, and the older the patient the more necessary its remembrance becomes.

### Treatment.

In the case before us the cause of the iritis is clearly defective elimination, with consequent auto-intoxication. The bowels were constipated, and the urine scanty, high-coloured, acid, with a specific gravity of 1028, and containing crystals of oxalate of lime. The retention of waste products in the blood as a result of auto-intoxication is a frequent predisposing cause of inflammation not only of the iris, but of every other structure of the body. Treatment, therefore, to be effective, must be both local and constitutional. The former is carried out by the use of atropine—a 1-per-cent. solution of the sulphate, which must be instilled repeatedly until full dilatation of the pupil is induced. Pain will be relieved most rapidly by aspirin in 15-grain doses every four or six hours. Copious douching of the conjunctival sac with hot saline solution will not only assist in relieving the pain, but will wash away the discharge with its micro-organisms. Bright light must be excluded from the eyes by the use of smoke-tinted glasses, but the patient should not be shut up in a dark room.

The general treatment should aim at promoting free elimination. The bowels and kidneys must be made to act with due regularity and efficiency, and to this end a saline laxative such as Carlsbad salts will be found most useful. A teaspoonful in a tumblerful of hot water should be taken slowly every morning before breakfast, and, in addition, at least two large draughts of hot water ought to be taken during the day. The patient should have a hot bath at bedtime, and, in order to promote perspiration, should sleep in a flannel nightdress. Exposure to cold should be carefully guarded against, and the food should be simple in kind and sparing in quantity. Vichy, or any mild alkaline table water, may be taken freely, but from alcohol in every form there must be the strictest abstinence.

Rapid improvement is likely to follow this treatment, but the greater your experience the less will you expect speedy disappearance of the symptoms in such cases. Careful observation will soon teach you the truth of the clinical aphorism that the longer a cause has been in operation the more intractable to treatment, and the more liable to recurrence, is the resulting disease. Acute symptoms may disappear quickly after the appropriate treatment has been begun, but the inflammation will always, even in the most favourable circumstances, take from three to six weeks to run its course. It is a wise rule, therefore, to exercise careful supervision over the patient, and to forbid the discontinuance of either the local or the constitutional treatment for a fortnight after all signs of inflammation have disappeared.

### A Case of Recurrent Iritis.

The second patient is a man of thirty-eight years of age, who was admitted to the institution on June 11, 1910. He says that twenty years ago he suffered from acute rheumatic fever, but has since that time enjoyed good health, save for repeated attacks of inflammation of the eyes, which have practically occurred once every year for the last six years. The right eye was attacked on every one of these occasions, but the left only twice. The seizures were very acute, and lasted from three to six weeks, and the character and severity of the symptoms were always much alike. The illness from which he is now suffering began twelve days before he came to the hospital. He is a bonded-warehouseman, and says that he is, when at work, much exposed to draughts of cold air, and that experience has taught him that if he gets his feet wet he is almost certain to suffer from eye inflammation. The first symptom is usually sudden pain in the eye affected, and in a few hours that is followed by great intolerance of light and by copious lachrymation. The pain steadily increases, and is, when at its worst, almost unbearable. It radiates from the eye to the forehead, temple, and cheek, and it is always most severe at night and during the early hours of the morning. The patient has then to rise from bed and walk about the room in a vain attempt to find relief, and, as a result of the sleeplessness consequent on the suffering, he soon feels very ill.

On the present occasion it is the right eye that is again attacked. There is very great circumcorneal injection, and the congestion of the deep vessels is easily distinguishable from the superficial redness of the conjunctiva. The cornea is quite transparent, the anterior chamber deep, the aqueous slightly turbid, the iris greatly discoloured, and the pupil contracted and irregular in outline. Perhaps, however, the most prominent symptom is the very intense tenderness revealed when pressure is applied over the ciliary region. The patient is a big, strong man, but, as you see, no sooner is the upper aspect of the eyeball touched through the closed lid than he pulls himself away and cries out with pain. The visual acuity is reduced to 6/60 in the right eye, and owing to the presence of floating bodies in the vitreous the details of the fundus are seen very indistinctly. The visual acuity of the left eye is practically normal, but a few posterior synechiæ tell of the two attacks of inflammation already mentioned. The intraocular tension is normal in both eyes.

### Points in Treatment.

The two most outstanding features here are the very great severity of the pain and the history of repeated attacks. The case might, indeed, be designated one of recurrent iritis. The predisposing cause is rheumatism. There is not only the clear existence of acute rheumatism twenty years ago, and of extreme sensitiveness to cold and damp, but the pain, which resisted repeated leeching and other remedies, disappeared as if by magic after the administration of thirty grains of salicylate of soda. Moreover, the urine was high in colour, acid, with a specific gravity of 1034, and contained numerous crystals of oxalate of lime. The treatment in this instance then must be modified to combat the rheumatic diathesis. Atropine, fomentations, and leeching are the principal local remedies that have been used, and, as has been said, salicylate of soda has been given for the alleviation of pain. Whenever in such cases the acute symptoms are relieved, a prolonged course of iodides and alkalies should be prescribed. The best guide is repeated and careful examination of the urine.

If the patient before you were a rich man, the proper course would be to send him to a spa, care being taken to select the most suitable locality and the waters best fitted for his individual requirements; but, whether the patient be rich or poor, a proper regimen is the most important factor in the treatment. Such patients as a rule eat too much and take far too little exercise; and consequently their habits and mode of life require to be very carefully considered. Their food should be plain and well cooked; and they should be on their guard against eating too much, while alcohol in all its forms must be entirely avoided. Daily exercise in the open air, short of fatigue, is absolutely necessary, and the dwelling-house, more especially the sleeping apartment, should be well ventilated. Marked improvement in health is sure to follow such careful regimen, and the eyes will share in the general well-being. Nor must the patient ever depart from the prescribed method of life, for if he weary of the restrictions imposed on him and return to the old state of things, the eyes will in all probability again become inflamed, and each fresh attack will be more and more difficult to cure.

*(To be continued.)*

